# Obituary

**Published:** 1869-06-01

**Authors:** James S. Whitmire

**Affiliations:** Metamora, Ill.


### OBITUARY.

DIED—JEROME BUCKNER EGBERT, M. D., son of Captain Louis J. Egbert, in Henry, Marshall county, Ill., May 14, 1869, aged 27 years, 5 months, 1 day. Dr. Egbert studied two years with Dr. J. W. Gordon, of Georgetown, Ohio, during which time he attended one course of lectures in one of the Ohio schools of medicine, in Cincinnati. When the war broke out in 1861, he enlisted in company C, 59th Regiment Ohio Volunteer Infantry, and was immediately made Hospital Steward at Camp Dennison, at which place he took sick and was discharged. In 1862 he enlisted as Hospital Steward of the

79th Ohio Volunteer Infantry, and served during the war in that capacity, going from Nashville to the sea, and at the end of the war received an honorable discharge. His father, in the meantime, having moved to Metamora Woodford county, Ill., Dr. Jerome turned his face westward, and, during the year 1865, spent the last year of his studentage in the office of Drs. J. S. and Z. H. Whitmire, in Metamora, Ill. During the winter of 1865–66, he attended his last course of lectures in Rush Medical College, Chicago, Ill., where, in the spring of 1866, he received the degree of Doctor of Medicine. In the spring of the same year he settled in Henry, Marshall county, Ill., in company with Dr. Charles Baker, an eminent practitioner, where he has remained ever since till his death. Dr. Egbert, during the three years in Henry, made himself an enviable reputation as a surgeon and practitioner of medicine. He was esteemed by the profession as a rising man and "a bright, particular star" in the field of science.

By the wealthy, he was esteemed as an estimable citizen; by the poor, he was reckoned their reliance in times of need; and by the philanthropist, he was esteemed for his many virtues and good qualities of head and heart.

When the world lost him, it was like a planet that has flitted from its orbit and ceased to shine upon the earth.

He will be taken to Georgetown, Ohio, and laid by the side of his sainted mother.

Peace be to his ashes, and may his virtues and example never fade from the memory of those who knew him. JAMES S. WHITMIRE.

Metamora, Ill., May 16, 1869.

